# Developing fit-for-purpose self-report instruments for assessing consumer responses to tobacco and nicotine products: the ABOUT™ Toolbox initiative

**DOI:** 10.12688/f1000research.16810.1

**Published:** 2018-12-02

**Authors:** Christelle Chrea, Catherine Acquadro, Esther F. Afolalu, Erica Spies, Thomas Salzberger, Linda Abetz-Webb, Stefan Cano, Benoit Arnould, Nelly Mainy, Jed Rose, Rolf Weitkunat

**Affiliations:** 1 PMI R&D, Philip Morris Products S.A., Neuchâtel, CH-2000, Switzerland; 2 Patient-Centered Sciences, Mapi, an ICON plc Company, Lyon, 69003, France; 3 Institute for Statistics and Mathematics, Institute for Marketing Management, Vienna University of Economics and Business, Vienna, 1020, Austria; 4 Patient-Centered Outcomes Assessments Ltd, Macclesfield, Cheshire, SK10 5LQ, UK; 5 Modus Outcomes, Letchworth Garden City, SG6 4ET, UK; 6 Rose Research Center, Raleigh, NC, 27617, USA

**Keywords:** Modified risk tobacco products, Reduced risk products, Self-report instruments, Behavior, Consumer perception, Best measurement practices

## Abstract

**Background.** Determining the public health impact of tobacco harm reduction strategies requires the assessment of consumer perception and behavior associated with tobacco and nicotine products (TNPs) with different exposure and risk profiles. In this context, rigorous methods to develop and validate psychometrically sound self-report instruments to measure consumers’ responses to TNPs are needed.

**Methods.** Consistent with best practice guidelines, including the U.S. Food and Drug Administration’s *“Guidance for Industry Patient-Reported Outcome Measures: Use in Medical Product Development to Support Labeling Claims,” *scientifically designed, fit-for-purpose, reliable, and valid instruments are now being applied to tobacco regulatory research.

**Results. **This brief report presents the ABOUT™ Toolbox ( **A**ssessment of **B**ehavioral **OU**tcomes related to **T**obacco and nicotine products) initiative. This communication: (1) describes the methodological steps followed for the development and validation of the measurement instruments included in the ABOUT™ Toolbox, (2) presents a summary of the high-priority tobacco-related domains that are currently covered in the ABOUT™ Toolbox (i.e., risk perception, dependence, product experience, health and functioning, and use history), and (3) details how the measurement instruments are made accessible to the scientific community.

**Conclusions. **By making the ABOUT™ Toolbox available to the tobacco research and public health community, we envision a rapidly expanding knowledge base, with the goals of (1) supporting consumer perception and behavior research to allow comparisons across a wide spectrum of TNPs, (2) enabling public health and regulatory communities to make better-informed decisions for future regulation of TNPs, and (3) enhancing surveillance activities associated with the impact of TNPs on population health.

## List of abbreviations

ABOUT: Assessment of Behavioral OUtcomes related to Tobacco and nicotine products;

FDA: U.S. Food and Drug Administration; ICF: International Classification of Functioning, Disability, and Health; mCEQ: Modified Cigarette Evaluation Questionnaire MRTP: Modified risk tobacco product; PRO: Patient-reported outcomes; PROQOLID: Patient-Reported Outcome and Quality of Life Instruments Database; RMM: Rasch measurement methods; TA: Translatability assessment; TNP: Tobacco and nicotine product; WHO: World Health Organization.

## Introduction

Many stakeholders have recognized that there is a risk continuum for tobacco and nicotine products (TNPs) ^1, 2^. On this continuum, combustible products, cigarettes in particular, present the most risk, because burning tobacco creates the vast majority of the harmful and potentially harmful constituents implicated in the development of smoking-related diseases ^3^. Cessation is at the lower end of the continuum, as it is the best way for smokers to lower their risk ^4^. Alternative noncombustible TNPs (sometimes referred to as alternative nicotine delivery systems) that avoid combustion lie somewhere between these anchoring points of the continuum ^1^. Tobacco harm reduction is an approach recognized by the U.S. Food and Drug Administration (FDA), the Institute of Medicine, and the World Health Organization (WHO) as part of a solution to more rapidly reduce the burden of preventable deaths and smoking-related diseases ^3, 5– 7^. In the U.S., this has given rise to a regulatory framework for manufacturers to market modified-risk tobacco products (MRTPs), defined by the FDA as “any tobacco product that is sold or distributed for use to reduce harm or the risk of tobacco-related disease associated with commercially marketed tobacco products” ^8^. However, to implement this approach successfully, consistent, transparent, and evidence-based science on the reduced risk potential of alternative products is paramount ^2^.

In alignment with the FDA’s draft guidance on MRTPs, consumer perception and behavior assessments are key components of assessing the full public health impact of tobacco harm reduction ^8^. Valid and reliable self-report measures are needed to assess consumer responses to MRTPs in comparison with other commercially available TNPs ^9– 11^. Although this has been acknowledged for quite some time ^10^, the field of tobacco regulatory research falls short of specifically developed measures due to the lack of adherence to measurement best practices and specific guidelines that would facilitate standardization and harmonization of measures across studies (e.g., see a recent review on risk perception measurement in tobacco control research ^11^). Some measurement and standardization initiatives have recently been proposed, the most predominant ones being the Patient-Reported Outcomes Measurement Information System Smoking Initiative ^12– 17^ and PhenX measures for Tobacco Regulatory Research ( https://www.phenxtoolkit.org/collections/trr). It is worth noting that both initiatives focus primarily on combustible tobacco products, with their development based on legacy measures developed solely for cigarettes ^15^. It is crucial, however, to attempt further efforts to develop new measurement instruments that would be “fit-for-purpose” to compare combustible and noncombustible products on the same risk continuum in order to better inform the public health decisions.

We present an ongoing collaborative effort to develop fit-for-purpose measurement instruments (i.e., concept-driven instruments providing interpretable outcomes for the purpose intended) to enhance the scientific framework of harm reduction. This new initiative has resulted in the creation of the ABOUT™ Toolbox (Assessment of Behavioral OUtcomes related to Tobacco and nicotine products).

The objectives of this communication are (1) to describe the methodological steps followed for the development and validation of the measurement instruments included in the ABOUT™ Toolbox, (2) to present a summary of the high-priority tobacco-related domains that are to be covered in the ABOUT™ Toolbox, and (3) to detail how the measurement instruments are to be made accessible to the scientific community.

## Methods

### Development and management of the ABOUT™ Toolbox

The ABOUT™ Toolbox has been developed using best measurement development practices and as the manifest of an underpinning behavioral conceptual model for TNPs.

Several guidelines, including the FDA’s “Guidance for Industry Patient-Reported Outcome Measures: Use in Medical Product Development to Support Labeling Claims” ^18^, have been used as the foundation for the creation of the ABOUT™ Toolbox initiative ^19, 20^. These guidelines provide the scientific basis for the development, modification, and validation of patient-reported outcome (PRO) measures in support of medical care research. Although not specifically designed for the tobacco regulatory research field, these recommendations are essential in outlining a wide range of development considerations, such as (1) defining the context of use and identifying the concept(s) of interest, (2) generating items that best capture the concept(s) of interest as expressed by the population of interest, (3) choosing the right response options and recall period, (4) evaluating the content validity of the instrument, and (5) assessing psychometric measurement properties for construct validity. These recommendations also cover the considerations around the adaptation of a self-report measure (e.g., for a different context of use, target population, or cultural group [see below the paragraph “cross-cultural equivalence”]).

The application of these best practices requires the use of mixed-methods research, which can be defined as “research in which the investigator collects and analyzes data, integrates the findings, and draws inferences using both qualitative and quantitative approaches, and methods in a single study or program of inquiry” ^21^. As noted in a recent paper on rare disease patients ^22^, qualitative methods alone are unable to inform us about the extent to which concepts are measurable. Conversely, quantitative methods alone cannot inform us about which concepts should be measured.

**Figure 1.**
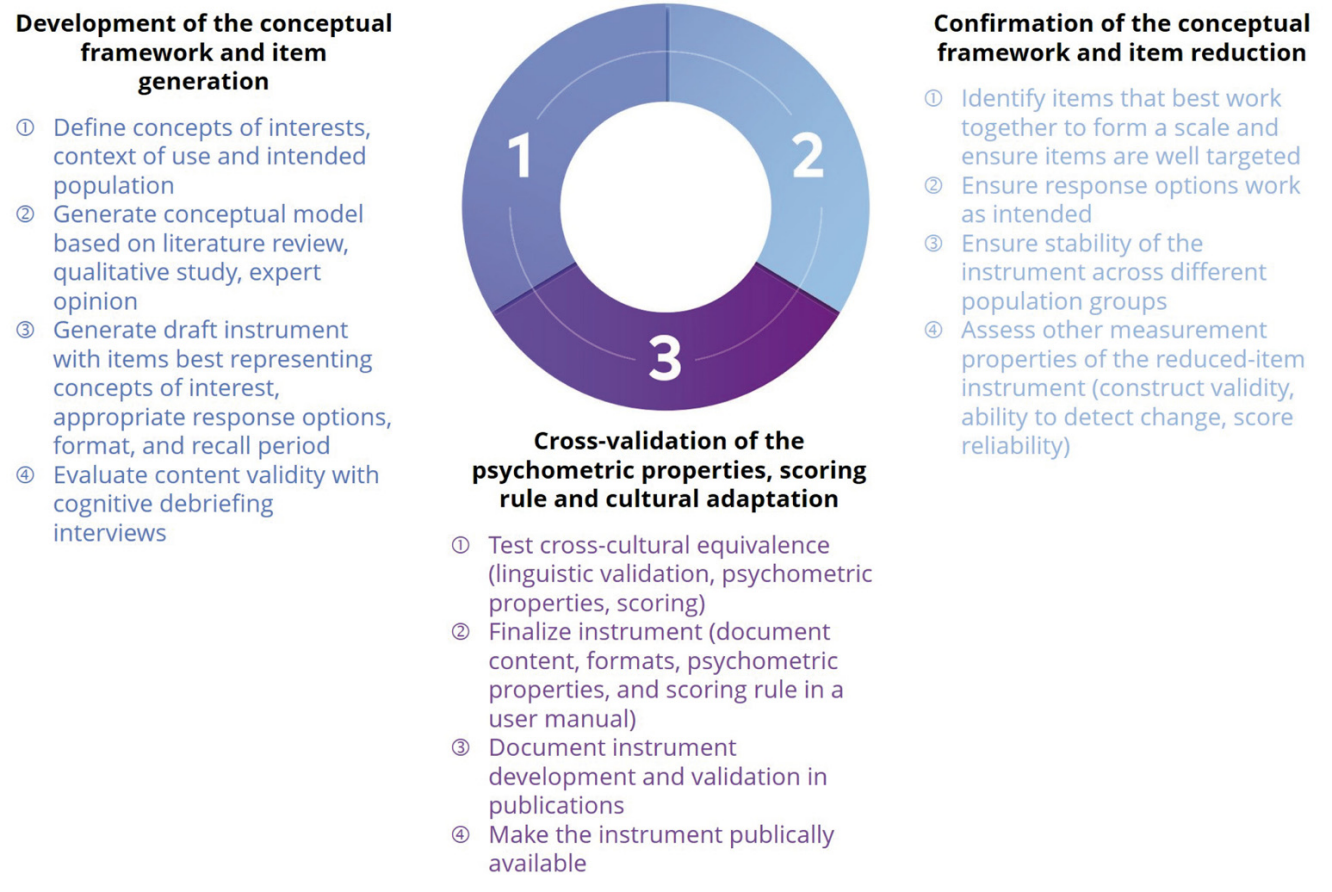
Iterative process for the development of an ABOUT™ instrument.

By applying these best measurement practices for the development of the ABOUT™ Toolbox (see Figure 1), the initiative further enhances tobacco regulatory research by combining five major components (described in the paragraphs below) that are paramount for rigorous instrument development.

**1. Generation of a conceptual framework**

The development of each instrument starts with the generation of a conceptual framework, which is grounded in theory and supported by the triangulation of evidence data from literature reviews, consumer input, and expert opinions. Furthermore, the development of each measurement instrument in the ABOUT™ Toolbox has been or is being conducted in close partnership with scientific experts from academic and commercial organizations with expertise in the fields of nicotine addiction, motivational aspects of consumer perception, and relevant areas on approaches to measurement (e.g., PROs, cross-cultural adaptation, psychometrics, regulatory submissions). The role of these experts is pivotal not only to provide input during the development of the conceptual framework but also to assist in the objective consensus-building process throughout the entire development of the instrument.

**2. Evaluation of content validity**

The main goal is to evaluate whether the instrument represents the concepts of interest, and the instructions and item content are appropriate, relevant, comprehensive, and understandable to the target population. This evaluation is performed in accordance with current good research practices ^23, 24^. The content validity of the instruments included in the ABOUT™ Toolbox is supported by the execution of qualitative research with consumers (e.g., 25– 27).

**3. Use of an appropriate psychometric model**

The psychometric assessment of most of the ABOUT™ instruments is based on the use of Rasch measurement methods (RMM) ^28^, supplemented by diagnostic evidence of the dimensionality and reliability properties rooted in classical test theory ^29, 30^. RMM particularly investigate to what extent (1) the items are targeted for the type and range of issues to be measured ^31^, (2) the items work well together as a set forming a unidimensional scale ^31– 33^, (3) the response options are working as intended ^34^, and (4) the items do not show differential item functioning across various population groups or TNPs ^35^. This evaluation provides the necessary evidence that the conceptual framework has been converted successfully into a list of items, the responses to which can be summed to form a statistically sufficient total score (i.e., comprising all available information). The score can then be transformed into a linear measure that is comparable across population groups or TNPs, conceptually meaningful and substantively interpretable (e.g., 36, 37). The final outcome of the application of RMM is the development of a calibrated scoring table that transfers sum scores to logit measures, which are mapped to a 0–100 scale for ease of interpretability. The conversion is a simple, linear transformation that changes the logit mean of 0 to 50 and converts most extreme measures to 0 and 100, respectively (e.g., 37). The purpose, description, administration, and scoring (including the calibrated scoring table) of the final validated version of an instrument is documented in a user manual that accompanies all the instruments of the ABOUT™ Toolbox.

**4. Cross-cultural equivalence of the ABOUT™ instruments**

**Figure 2.**
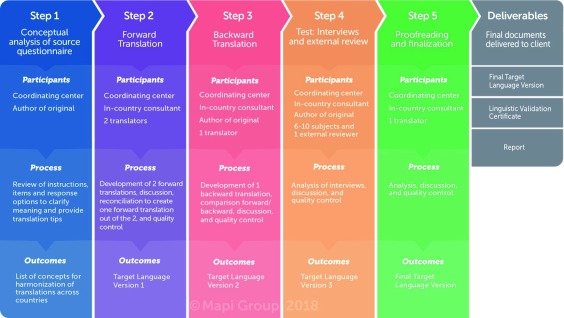
Linguistic validation process for the development of an ABOUT™ instrument.

In the context of the globalization of tobacco regulatory research, measures appropriate for use in different cultures are crucial. The demonstration of cross-cultural equivalence requires investigating that the instrument measures the same concepts in a comparable way across different languages and cultures ^38– 40^. The first step toward cross-cultural equivalence is to ensure that rigorous translation procedures are followed. In the field of health outcomes research, recommendations to support the use of self-reported measures in multinational contexts have been provided ^41, 42^. These recommendations apply to measures developed in one language and subsequently translated for use in other countries and cultures. In line with this guidance, the instruments included in the ABOUT™ Toolbox follow a thorough linguistic validation process consisting of two forward translations to the targeted new language, one back translation to the source language, and qualitative cognitive debriefing interviews with participants in the targeted language to ensure that the translations are understood (e.g., 43 and Figure 2) ^44^.

The investigation and demonstration of the cross-cultural equivalence of the ABOUT™ instruments are completed by quantitative steps (e.g., 37, 45), such as the evaluation of the psychometric properties of the translations and differential item functioning ^46^.

Translatability assessment (TA) is a technique that will be applied to all future ABOUT™ instruments. It is defined as the review of an original measure, preferably during the development stage, prior to its use, in order to determine its suitability for future translations in multilingual studies ^47^. TA can be viewed as the very first step toward ensuring measurement equivalence between the original measure and its future translations.

**5. Appropriate access and use of the validated instruments (original and translations)**

Easy, centralized, and appropriate access to original instruments and their translations is often a prerequisite to efficient research. With a unique point of access endorsed by the developers of instruments, researchers are able to access and use the original instrument and its translations ^48^. The centralization and control of access also enables the integrity of the each measurement instrument (original and translations) to be respected ^49^. Once the instruments are fully developed and validated (Steps 1 and 2 on Figure 1), they are made publicly available through the Patient-Reported Outcome and Quality of Life Instruments Database (PROQOLID™) ^50^, managed by Mapi Research Trust and part of the ePROVIDE™ web platform ( https://eprovide.mapi-trust.org/).

To get access to the instruments distributed on ePROVIDE™, each new user has to complete a free registration form at https://eprovide.mapi-trust.org/login (click to the Free Registration button). The following link leads to a tutorial to help navigating through the whole registration process: https://eprovide.mapi-trust.org/tutorials/registering-for-free. Once the registration is completed, the register button will lead the user to his/her free ePROVIDE™ account. From there, any retrieval of instruments can be performed.

Instruments and their respective user manuals can be retrieved through the main search engine of the database by using either the full name or parts of the name (e.g., ABOUT™–Perceived Risk, Perceived, or Risk) or within the “Our Catalog” section of ePROVIDE™ ( https://eprovide.mapi-trust.org/catalog) by filtering through the Behavior and Behavior Mechanisms category.

To be able to use any of the ABOUT™ Toolbox instruments, all users (whether they are commercial or non-commercial users) will have to accept the conditions of a license/user agreement and complete the appropriate form. This license, issued by Mapi Research Trust, which is the official distributor of the instruments, specifies the terms under which each instrument should be used: Special terms, i.e., specific to each instrument, and General terms: https://eprovide.mapi-trust.org/user-agreement-general-terms.

Tutorials are available on how to submit requests (commercial users) or download copies directly (non-commercial users) (see https://eprovide.mapi-trust.org/faq). All requests are handled by the PROVIDE team of Mapi Research Trust.

## Results

### Inventory of the ABOUT™ Toolbox

The ABOUT™ Toolbox currently comprises five measurement instruments, which are at various stages of development and validation. Table 1 presents a summary of the relevant domains, concepts of interest, and context of use to be covered by the instruments. The initiative is to be expanded by additional domains as they are identified. The initial inclusion of these current instruments were informed extensively by existing research and domains that have been prioritized based on public health impact and issues of key importance to tobacco regulatory research.

**Table 1.** Information on the ABOUT™ Toolbox and access to the instruments.

Instrument	Concept of interest (# items)	Context of use	Target population	Accessibility/Publications
**Fully developed and validated instruments**				
**Perceived** **Risk**	Health risk (18) Addiction risk (7) Harm to others (2) Social and practical risk scales are currently under development	All TNPs + Cessation	Adult current, former, and never TNP users	Available in PROQOLID™ under ABOUT™–Perceived Risk: https://eprovide.mapi-trust. org/instruments/about-perceived- risk-formally-perceived-risk- instrument-pri ^25, 37^
**Use History**	Initiation Cessation Intensity of current and past use	All TNPs	Adult current, former, and never TNP users	Available in PROQOLID™ under Smoking Questionnaire (SQ): https://eprovide.mapi-trust. org/instruments/smoking- questionnaire2 ^52, 53^
**Instruments in development**				
**Product** **Experience**	Satisfaction (3) Psychological reward (5) Craving reduction (1) Aversion (2) Enjoyment of respiratory tract sensation (1)	All TNPs Different recall periods	Adult current TNP users	Available in PROQOLID™ in 2019
**Dependence**	Time to first and last product use (2) Attitudinal evaluation (5) Behavioral evaluation (5)	All TNPs	Adult single or poly-TNP users	Available in PROQOLID™ in 2019
**Health and** **Functioning**	Body structure and function Activity Participation Personal factors Environmental factors	All TNPs + Cessation	Adult current and former TNP users	Development initiated in 2018

PROQOLID™, Patient-Reported Outcome and Quality of Life Instruments Database; TNPs, Tobacco and Nicotine Products.

### Fully developed and validated instruments

***1. ABOUT™−Perceived Risk***

Assessment of risk perception is an important domain of tobacco-related behaviors and influences any tobacco harm reduction strategies aimed at getting individuals to switch to less harmful alternatives to cigarette smoking ^1, 11^.

The Perceived Risk Instrument was developed as a multiscale instrument intended to assess the perceived risks associated with use across a range of different of TNPs, relative to other products, cessation aids, and quitting all tobacco products. The health risk and addiction risk scales have been calibrated in three countries (U.S., Italy, and Japan) for two perception types: risk to the individual respondent (personal risk) and risk to users of the product in general (general risk) ^25, 37^. The validation of further scales for perceived social and practical risk is currently under development.

***2. ABOUT™−Use History***

One of the key aims underlying tobacco control and harm reduction is to reduce the burden of smoking-related diseases through the implementation of monitoring strategies for tobacco consumption and characterization of key patterns and trends in tobacco use ^3^. The Smoking Questionnaire, included in the ABOUT™ Toolbox, was developed to provide a core set of questions that cover the major dimensions of TNP use and is consistent with criteria used for defining smoking history and status ^51^. It assesses frequency and intensity of current and past TNP use behavior, initiation, and cessation and demonstrates good test-retest reliability ^52, 53^.

### Instruments under development

***1. ABOUT™−Product Experience***

Product experience encompasses a range of self-reported expressions of an individual’s experience using a TNP and is a key predictive measure of short-term preference and long-term TNP use ^54^. The modified Cigarette Evaluation Questionnaire (mCEQ) has been endorsed by regulatory and public health bodies to use in the context of MRTP assessment ^9, 55^. The ABOUT™ Toolbox includes a measure consisting of two multi-item scales and three single-item scales arising from an adaptation and rewording of the mCEQ ^56^ and the Product Evaluation Scale ^57^. The scales focus on satisfaction, psychological reward, craving reduction, aversion, and enjoyment of respiratory tract sensation. Psychometric testing and validation have been carried out for the use of the measure to assess cigarettes and heat-not-burn products ^45^, and further validation is ongoing for a variety of TNPs (e.g., e-cigarettes, cigars/cigarillos, smokeless products) and different recall periods.

***2. ABOUT™−Dependence***

Nicotine dependence has been shown to be a primary driver of smoking and TNP use behaviors ^58^. However, dependence on nicotine has generally focused on cigarette smoking. Evidence suggests that measuring dependence with a common set of symptoms across different TNP use groups is feasible and may better reflect the dynamics of dependence across a range of products ^59– 61^. Work is ongoing to develop such a fit-for-purpose dependence instrument for inclusion in the ABOUT™ Toolbox. The proposed instrument is rooted in a conceptual framework of dependence that identifies lack of control (e.g., urgency to use upon waking up, difficulty to cease using, self-awareness of dependence) as the core construct. Recommended practices in PRO development have been used to generate a draft instrument from a range of qualitative research steps, including literature review, concept elicitation, and cognitive debriefing interviews with different groups of adult tobacco users ^27^. The draft instrument is currently undergoing quantitative field testing to assess psychometric properties and produce a final validated measure.

***3. ABOUT™−Health and Functioning***

Health and functioning is a relevant dimension for the evaluation of TNP impact on public health and requires further investigation ^8, 9, 58^. There are no established measures specific to tobacco-related health outcomes to date. In addition, existing generic health and functioning instruments do not capture the small, but potentially important, concepts that may change when a smoker switches to an MRTP. Currently, efforts are ongoing to develop a new outcome measure for inclusion in the ABOUT™ Toolbox that would accurately reflect the health and functioning status of individuals who use TNPs, with a particular focus on healthy adult smokers who switch to MRTPs. The measure’s development will be underpinned by conceptual frameworks including, but not limited to, the WHO’s International Classification of Functioning, Disability, and Health (ICF) ^62^ and the Revised Wilson and Cleary Model for Health-Related Quality of Life ^63^. The ICF is particularly well suited to serve as a guiding framework, as it conceptualizes a person’s level of functioning as a dynamic interaction between body structure and functions, health conditions, social participation, personal factors, and environmental factors. The Wilson and Cleary Model is included to address subjective dimensions of health and functioning, such as well-being and health-related quality of life. Literature review, an expert panel, and qualitative research with a wide range of consumers are planned for the initial phase of the project.

## Discussion and Conclusion

In the present paper, we have presented a new initiative aiming at enhancing the scientific framework of harm reduction and promoting the establishment of consensus and standardized tools to be used across tobacco regulatory research studies. Within the new regulatory MRTP pathway, FDA can issue an order authorizing the marketing of MRTPs. To do so, data must demonstrate that use of the new product (1) would present a significantly lower risk of harm to the individual user and (2) would reduce the incidence of harm in the population as a whole (e.g., with evidence that marketing the new product as a less risky alternative to cigarettes would not increase use of the new product by non-smokers), as described in section 911 of the Federal Food Drug and Cosmetic Act ^64^. Since the inception of the MRTP pathway in 2011, FDA has received 35 MRTP applications and granted zero Modified Risk Orders ( https://www.fda.gov/TobaccoProducts/Labeling/TobaccoProductReviewEvaluation/ucm304465.htm#2). This suggests that (1) no manufacturer has yet successfully demonstrated the harm reduction potential of any new TNP to the FDA and (2) the FDA and manufacturers have not shared a common understanding of what classifies consistent, transparent, and evidence-based science. Therefore, it is of paramount importance to establish a common, well-defined understanding of the types and volume of research data that would demonstrate a candidate MRTP to be appropriate for the protection of the public health to the FDA and other regulatory authorities. As expressed by the National Institutes of Health Tobacco Regulatory Science Program and the FDA Center for Tobacco Products, “One way to accomplish this is to provide investigators with a common set of tools and resources to facilitate sharing, comparing, replication of findings, and integration of data from multiple sources” ^65^.

As part of this effort, the current ABOUT™ Toolbox may facilitate progress toward consensus of domains to be assessed and consensus on how they should be measured and reported. With the development and dissemination of the ABOUT™ Toolbox, researchers will have access to instruments that are (1) developed and validated with state-of-the-science methods to be psychometrically sound, straightforward to implement in clinical and population-based studies, and easy to interpret; (2) created to be relevant and applicable across the whole spectrum of TNPs and across various population groups; and (3) designed to enhance standardization and comparison of data on perception and behaviors toward MRTPs across academic, industry, and public health research communities.

The current measurement instruments highlighted in the ABOUT™ Toolbox fit within what can be described as a broader behavioral conceptual model, designed to understand TNP switching or transition behaviors, which encompasses several levels of assessment (i.e., individual, product, and environment [Spies *et al*., manuscript in preparation]). This conceptual model evolved from the review of several existing frameworks that propose explanations of and factors associated with TNP use ^10, 66, 67^ and was complemented by literature on principles of behavioral changes, taking a socio-ecological approach to the conceptualization of TNP switching or transition behaviors ^68^. Each of the individual, product, and environment levels includes several categories for which concepts and variables are defined. For instance, the individual level includes individual traits (e.g., dependence), attitudes and beliefs toward products (e.g., perceived risk), response to the product (e.g., satisfaction), self-reported product behavior (e.g., consumption changes), and functional health and quality of life. Instruments within the ABOUT™ Toolbox are intended to be used within this conceptual model to measure each of these concepts of interest in a standardized way.

By making the ABOUT™ Toolbox available to the tobacco research and public health communities through PROQOLID™, we envision a rapidly expanding knowledge base, with the goals of not only advancing further the interpretation of consumer perception data comparing a large spectrum of TNPs but also enabling public health and regulatory communities to make better-informed decisions for future regulation of MRTPs and to enhance surveillance activities associated with smoking-related disease. The ABOUT™ Toolbox launches a dialogue on new perspectives required to develop standards and best practices in the spirit of current guidance for self-reported measures and may encourage the creation of a consortium to work on standard measures across the industry.

## Data availability

Access to the measurement instruments, as well as further information on the original versions and translations is freely available for non-commercial use on ePROVIDE™ ( https://eprovide.mapi-trust.org).
