# Supplementary material for: Developing fit-for-purpose self-report instruments for assessing consumer responses to tobacco and nicotine products: the ABOUT™ Toolbox initiative
Source: F1000Res. 2018 Dec 2;7:1878. [Version 1] doi: 10.12688/f1000research.16810.1 (PMC6415329; doi:10.12688/f1000research.16810.1)
Supplement: Peer review history

# Peer review: Reviewer response for version 1

**DOI:** 10.5256/f1000research.18377.r44881

**Published:** 2019-03-08

**Authors:** Michael Balls, Robert D. Combes

**Affiliations:** 1 University of Nottingham, Nottingham, UK; 2 Independent Consultant, Norwich, UK

This article deals with an important issue facing society today – how to encourage tobacco harm reduction without introducing new and undefinable hazards, and to make possible the perception of risk by individuals using a range of devices. While not wishing to question the integrity of the authors or the value of the thought and effort they have put into developing ABOUT ^TM ^Toolbox, we think it essential that wider questions related to the development of safer smoking materials and alternative sources of nicotine are considered, before turning to specific aspects of the ABOUT ^TM ^Toolbox proposal itself.

 In the Introduction, the authors refer to the continuum between cigarette smoking at one extreme and cessation at the other. However, it is not clear whether “cessation” refers only to tobacco products, including non-combustible TNPs, or to all forms of voluntary uptake of nicotine. Our own position, as toxicologists, is clear. Nicotine is a very toxic and addictive substance, so its use should be actively discouraged. Therefore, the increasing use of electronic cigarettes (ECs) is a matter of great public health concern.

 Another continuum is that between heavy cigarette smokers and individuals who have never smoked at all. Encouraging heavy smokers to switch to ECs in order to reduce their risk of tobacco harm can be a helpful strategy, but encouraging non-smokers to use ECs can only be seen as highly undesirable.

 Public Health England (PHE), an executive agency of the Department of Health and Social Care, has stated that ECs are 95% safer than tobacco cigarettes. Such a statement is without scientific justification, as there are no compatible quantitative measures of the risks involved in smoking cigarettes or ECs, which would permit such a comparison between them to be made. The statement is based on the conclusions of a small number of experts who advise PHE.

  Safety is related to risk, which is the product of hazard and exposure. However, there is no satisfactory body of scientific evidence concerning the hazard represented by the vapours produced by ECs. The effects of exposure to EC vapour must also be highly variable, as they will depend on a variety of factors, including the type of EC used, the sources and levels of its nicotine and other additives, puff volume, puff frequency, the extent of inhalation, and the age, previous smoking history, health conditions affecting the respiratory system, such as asthma, and genetic polymorphism in relation to, for example, nicotine biotransformation enzymes, and nicotinic-acetylcholinergic receptor subunit structures, which affect binding affinity for nicotine. In combination, these and other factors must mean that there is also a continuum of individuals subject to high to low risks from exposure to EC vapour, whatever those risks may be.

 An important principle in society is that individuals should have the freedom to make their own decisions about what they do, bearing in mind the risks involved, as they are able to perceive them. However, given the lack of evidence about the safety of alternatives to cigarette smoking, how can members of the general public be expected to make their own decisions about what, if anything, to smoke, and with what limitations?

 The article refers to a triangulation of evidence from literature reviews, consumer input and experts opinions, but we would argue that none of these are a satisfactory basis for the self-assessment of risk. Such as it is, the literature is sparse and conflicting, and both consumers and policy makers are strongly influenced by the media, where reporters are easily led by soundbites, such as the PHE “95% safer” slogan. In addition, finding experts without conflicts of interest is not easy. There is a continuum from individuals who lack sufficient expertise to those who have a great deal of expertise, but those at the latter end of the spectrum are likely to have conflicts of interest as a result of the ways in which their experience was gained. We note that the list of experts mentioned in relation to the generation of a conceptual framework did not include toxicologists. Having a sufficient basis for perceiving the potential toxic effects of tobacco and other nicotine products is surely as essential to ABOUT ^TM ^Tool as is the use of an appropriate psychometric model.

 The development of systems such as ABOUT ^TM ^Toolbox raises many important questions, including how, and by whom, they are designed and managed, the results are analysed and reported, and their outcomes are used in making policy decisions. This must involve high degrees of independence and transparency, and there is also a continuum in terms of motivation. At one end are those responsible for advising on public health, while at the other end are those with commercial interests in developing and maintaining markets and dividends. These various motivations are defensible, and those with one kind of responsibility should recognise the responsibilities of others placed elsewhere in the continuum.

 This leads us to other matters of particular concern. First, the move from cigarettes smoking to the use of ECs should only be a stage in a longer process, leading to the avoidance of all forms of nicotine, however, consumed, in order to protect the delicate tissues of the respiratory system, in particular, from avoidable harm resulting from exposure to toxic and addictive substances. There is growing evidence that that is not a scenario that the tobacco industry wants, as the industry appears to be aiming to replace one kind of profitable dependence on addiction to nicotine with another one, on the grounds that it is less harmful. Secondly, tobacco harm results from repeated and long-term exposure to tobacco smoke, but there is no available evidence concerning the long-term effects of repeated exposure to EC vapours. Here, then, the precautionary principle should apply, and, ideally, the health of cohorts of EC users and non-users should be monitored over several years, before any statements about safety are made. Thirdly, we are all taken in by the tactic of pricing of goods at $10.99, which seems lower than $11.00. Similarly, we tend to think that, if something is 95% safer than something else, it is absolutely safe. That is not true for ECs. This tendency may be useful to the short-term ambitions of PHE and to the commercial hopes of the producers of ECs, but cannot possibly be a satisfactory and acceptable basis for a public health policy. Fourthly, there must be a clear distinction between policies aimed and assisting cigarette smokers to switch to something less harmful and those aimed at individuals who have never smoked at all. Regrettably, there are campaigns aimed at attracting young people, including children, to use ECs, and there is evidence that individuals who begin to take in nicotine via ECs, later switch to cigarettes.  

 We hope that the authors may wish to consider our points and perhaps make some minor modifications to their article.

We have read this submission. We believe that we have an appropriate level of expertise to confirm that it is of an acceptable scientific standard.

# Peer review: Reviewer response for version 1

**DOI:** 10.5256/f1000research.18377.r43288

**Published:** 2019-02-12

**Authors:** Christopher Proctor

**Affiliations:** 1 R&D Centre, British American Tobacco (Investments) Ltd., Southampton, UK

The authors tackle an important issue in tobacco regulatory science - how to integrate data on the potential impact of the introduction of potentially reduced risk tobacco and nicotine products on the population. It draws on approaches to labelling claims on medical products, and as such may miss some important elements of what may influence behaviours and use of what are in most jurisdictions consumer products. Some of these may become apparent as the tool is used in a multi-national context. For example, the cost of the devices of vaping or 'heat-not-burn' may have a major influence on the likely uptake in countries with low disposable income.  Other cultural influences may also become important, and so rather than a translation followed by back translation, additional items may need to be added in some countries.

I have read this submission. I believe that I have an appropriate level of expertise to confirm that it is of an acceptable scientific standard.
